# New Insights on the Electronic-Structural Interplay in LaPdSb and CePdSb Intermetallic Compounds

**DOI:** 10.3390/ma15217678

**Published:** 2022-11-01

**Authors:** Matthias Josef Gutmann, Gheorghe Lucian Pascut, Kenichi Katoh, Martin von Zimmermann, Keith Refson, Devashibhai Thakarshibhai Adroja

**Affiliations:** 1 Science and Technology Facilities Council, Harwell Campus, ISIS Facility, Chilton Didcot, Oxfordshire OX11 0QX, UK; 2 MANSID Research Center, University Stefan Cel Mare, 720229 Suceava, Romania; 3 Department of Applied Physics, National Defense Academy, Yokosuka 239-8686, Japan; 4 Deutsches Elektronen-Synchrotron DESY, 22607 Hamburg, Germany; 5 Highly Correlated Matter Research Group, Physics Department, University of Johannesburg, P.O. Box 524, Auckland Park, Johannesburg 2006, South Africa

**Keywords:** intermetallic LaPdSb and CePdSb compounds, crystal structures, single-crystal neutron and X-ray scattering, experimental and simulated diffuse scattering, DFT and DFT+ embedded DMFT, electronic properties, Fermi surface

## Abstract

Multifunctional physical properties are usually a consequence of a rich electronic-structural interplay. To advance our understanding in this direction, we reinvestigate the structural properties of the LaPdSb and CePdSb intermetallic compounds using single-crystal neutron and X-ray diffraction. We establish that both compounds can be described by the non-centrosymmetric space group P6_3_mc, where the Pd/Sb planes are puckered and show ionic order rather than ionic disorder as was previously proposed. In particular, at 300 K, the (h, k, 10)-layer contains diffuse scattering features consistent with the Pd/Sb puckered layers. The experimental results are further rationalized within the framework of DFT and DFT+ embedded DMFT methods, which confirm that a puckered structure is energetically more favorable. We also find strong correspondence between puckering strength and band topology. Namely, strong puckering removes the bands and, consequently, the Fermi surface pockets at the M point. In addition, the Pd-d band character is reduced with puckering strength. Thus, these calculations provide further insights into the microscopic origin of the puckering, especially the correspondence between the band’s character, Fermi surfaces, and the strength of the puckering.

## 1. Introduction

RPdSb (R = Rare Earth) compounds have attracted a lot of interest over the years due to their novel and multifunctional physical properties, such as anisotropic magnetic behavior with high ordering temperature [1,2,3], quasi-two-dimensional conductivity [4,5,6], Kondo behavior combined with ferromagnetic order [1,2,3,7], half-metallicity within the ferromagnetic ground state [8,9,10], unusual and surface electronic states [11,12], thermoelectricity [13,14], and non-centrosymmetric crystal structure [15]. Out of these compounds, a lot of attention was given to CePdSb due to its possibility of being a Kondo-lattice system with ferromagnetic ordering [1] and to LaPdSb for its potential thermoelectric properties [13,14]. Systematic powder diffraction studies of the RPdSb crystal structures showed that these materials crystalize in three structure types, hexagonal CaIn_2_-type (P6_3_/mmc No. 194), orthorhombic TiNiSi-type (Pnma No. 62), and cubic MgAgAs-type (F-43m No. 216), depending on the type of R ion [16]. Initially, CePdSb and LaPdSb were assigned a structural model of hexagonal CaIn_2_-type, where the R ions occupy the 2(b) Wykoff positions and the Pd and Sb ions are randomly distributed over the 4(f) crystallographic sites [3,16]. Further X-ray powder diffraction studies showed that LaPdSb is better described by the ZrBeSi-type structure, the same hexagonal CaIn2-type but with ordered Pd and Sb ions (La, Pd, and Sb occupy the 2(a), 2(c) and 2(d) Wykoff positions) [9,13,14]. Powder neutron diffraction studies combined with crystal field excitations were used to propose a new model for CePdSb described by a slight modification of the hexagonal CaIn_2_-type structure where the Pd and Sb are ordered and puckered within the Pd/Sb layer (hexagonal GaGeLi-type structure, P6_3_mc, No. 186). Neutron powder diffraction alone could not distinguish between disordered P6_3_/mmc and ordered P6_3_mc because the neutron scattering lengths of Sb and Pd are very similar [2,17]. Thus, so far, we have found in the literature that single-crystal X-ray diffraction [5,6] and powder neutron diffraction [2] point toward the P6_3_mc space group with ordered Pd and Sb ions, while single-crystal X-ray diffraction [4], powder neutron diffraction [2], and powder X-ray diffraction [1,3,10] point toward P6_3_/mmc space group with ordered/disordered Pd and Sb ions. In addition, XPS measurements combined with first-principles calculations point toward the preference for Pd and Sb ionic ordering [8].

**Figure 1:**
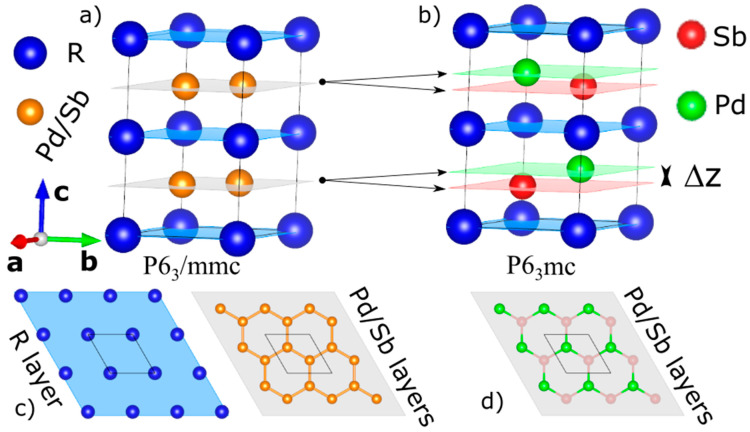
Crystal structures for RPdSb, R-La, and Ce: (**a**) crystal structure associated with space group P6_3_/mmc (model 1), CaIn_2_-type structure; (**b**) crystal structure obtained from a joint refinement of the neutron and X-ray single-crystal data associated with space group P6_3_mc (model 3), ZrBeSi-type structure; (**c**,**d**) schematic representation of the R, Pd, and Sb alternating layers; the black double arrows show the splitting of the disordered Pd/Sb layers into two ordered Pd and Sb layers by an amount Δz; blue, grey, red, and green sheets show schematically the atomic layers.

In order to shed light on the correct crystal structure model for the LaPdSb and CePdSb compounds, we have performed single-crystal neutron and X-ray diffraction studies with additional first-principles calculations in the form of density functional theory (DFT) and density functional theory with embedded dynamical mean field theory (DFT+eDMFT). The current neutron and X-ray single-crystal diffraction studies show that the space group P6_3_mc provides a better description of the crystal structure, resulting in a puckering of the Pd/Sb layers and ionic ordering within these layers (see Figure 1). Neutron diffuse scattering shows a clear signature of the ordering and puckering of the Pd/Sb ions within layers. Total energy calculations for both compounds show that the crystal structure models described in the P6_3_mc space group are energetically more stable than the previously assigned models described by the P6_3_/mmc. Furthermore, atomic and orbital projected electronic band structure calculations give hints towards the electronic-structural interplay; in other words, towards the stability of the model described by the P6_3_mc versus the P6_3_/mmc space group. Our DFT+eDMFT calculations are validated by the qualitative agreement with previously reported XPS measurements [10,11].

## 2. Methods

Experiments: Single crystals of LaPdSb and CePdSb intermetallic compounds were grown by the Bridgman technique using tungsten crucibles with starting materials for the synthesis, of 99.9% purity for Ce and La, 99.98 purity for Pd and 99.999% purity for Sb. Further details of the single-crystal synthesis are given elsewhere [6]. Using this technique, we were able to obtain single crystals of a few mm^3^ (cubic form with each side of about 5 mm). These crystals were found to slowly decompose over time when exposed to air for prolonged periods; hence, they were kept either in a vacuum or noble gas atmosphere, such as Ar or He.

Several pieces were tested, and full data sets were collected on LaPdSb and CePdSb at room temperature using the single-crystal diffractometer SXD at the ISIS spallation neutron source [18]. For the experiment, a single crystal was attached to an Al pin using thin strips of adhesive Al tape. The sample was mounted on a closed-cycle refrigerator inside the evacuated sample tank. Relatively long exposures were taken at 6–10 different crystal orientations with the aim of capturing both the crystal structure and diffuse scattering in-between the Bragg peaks. Data reduction was performed using the in-house software SXD2001 [19] to obtain structure factors and reciprocal space sections. Crystal structures were solved using the JANA2020 program and the charge-flipping algorithm [20].

A complementary dataset was recorded for LaPdSb using high-energy X-rays on beamline BW5 at DORISIII at DESY (Hamburg, Germany) with an X-ray energy of 100 keV and a Perkin Elmer XRD1621 flat panel detector (Fremont, CA, USA). During this experiment, the crystal was rotated around a vertical axis in steps of 0.25 degrees, covering an angular range of 180 degrees, corresponding to 720 images. The same scan was repeated 5 times. Structure factors were obtained using the program XDS [21]. Reciprocal space sections were reconstructed using an in-house code. Detector artefacts due to blooming around saturated pixels and streaking during readout were treated similarly to [22].

**Figure 2:**
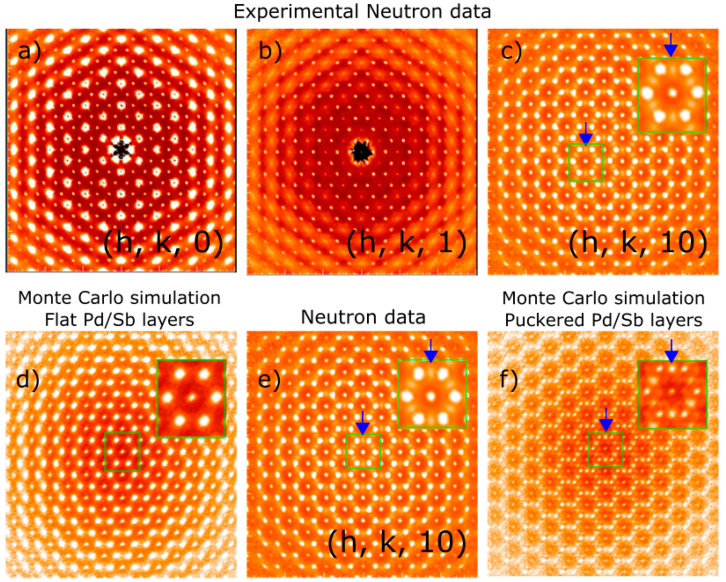
Diffuse scattering for CePdSb: panels (**a**–**c**) show experimental diffuse scattering for (h, k, 0), (h, k, 1), and (h, k, 10) layers; panels (**d**–**f**) show the simulated diffuse scattering from the balls-and-springs Monte Carlo simulation corresponding to flat Pd/Sb (model 1) and puckered layers (model 3); blue arrows show the presence of extra features for (h, k, l) layers with large l, panel (**c**)-data and highlights the features in the diffuse scattering associated with the presence of puckering, panel (**f**)-simulation (Bragg peaks have been subtracted in the MC simulation but not the data); insets show zooms of the region inside the green rectangle.

**Figure 3:**
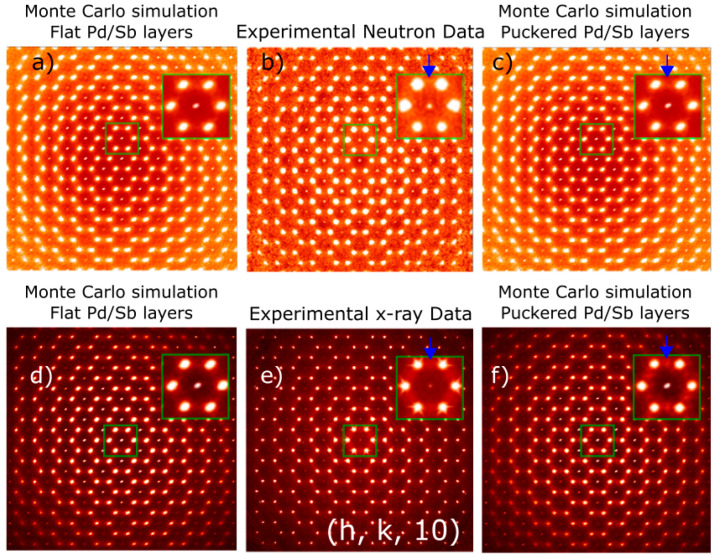
Diffuse scattering for LaPdSb: panels (**b**,**e**) show neutron and X-ray experimental diffuse scattering for (h, k, 10) layer; panels (**a**,**c**,**d**,**f**) show the simulated diffuse scattering from the balls-and-springs Monte Carlo simulation corresponding to flat Pd/Sb and puckered layers; blue arrows show the presence of experimental features and highlight these features in the diffuse scattering associated with the presence of puckering (Bragg peaks have been subtracted in the MC simulation but not the data); insets show zooms of the region inside the green rectangle.

Monte Carlo Simulation for Diffuse Scattering: Monte Carlo (MC) simulations were carried out using a simple balls-and-springs model for flat and puckered Pd/Sb layers. A model crystal of size 48 × 48 × 48 unit cells was used with periodic boundary conditions to compute diffuse scattering with a resolution of 1/48 reciprocal lattice units. Springs were attached between nearest-neighbors Ce-Pd, Ce-Sb, and Pd-Sb, and a model similar to what has been presented previously in the literature [23] was used to model diffuse scattering. One MC cycle corresponds to 6 × 48 × 48 × 48 moves, which is the total number of atoms in the model crystal, such that each atom is visited once on average. 200 MC cycles were found to be sufficient for convergence towards the target value of thermal displacement parameters obtained from the crystallographic refinements. In each move, an atom is chosen randomly and displaced by a random amount in a random direction. An energy expression of the form of Hooke’s law is used to compare the energy of the configurations before and after the move and accepted or rejected accordingly. In the latter case, rejection is based on drawing a random number in the interval 0 to 1 and compared with exp(−ΔE/k_B_T), with ΔE being the energy difference between the energy configurations after and before the move and k_B_T = 1.0. If the random number is smaller than the Boltzmann expression, then the move is still accepted; otherwise, it is being rejected. This energy expression is as follows:

$$E_{MC}=\sum^{\;}k_{i}{\left(d_{i}-d_{0,i}\right)}^{2}$$

where $k_{i}$ the force constant for bond *i* and $d_{i}$ and $d_{0,i}$ the current and equilibrium bond length, respectively. After much trial and error, a ratio of the force constants of 1:1:1 was found to be satisfactory and to reproduce the diffuse scattering patterns qualitatively in great detail. Inclusion of further distant neighbors necessitated the corresponding force constants to be at least 50 times weaker than the nearest-neighbor ones; thus, we considered these effects to be too small and neglected them. Diffuse scattering patterns from this model crystal were calculated using 200 lots of size 10 × 10 × 10 unit cells with the program described in [24]. Results of the MC simulations are shown in Figure 2d,f for CePdSb and in Figure 3a,c,d,f for LaPdSb.

First-principles calculations: Density functional theory (DFT) and density functional theory plus embedded dynamical mean field theory (DFT+eDMFT) calculations were performed in order to understand the electronic properties of these compounds. DFT calculations were performed with the wien2k code, which implements a self-consistent all-electrons full-potential linearized augmented plane wave method to solve the Kohn–Sham equations [25]. Within this method, the unit cell volume is decomposed into two regions: (I) non-overlapping muffin-tin (MT) spheres (of radius R_MT_) centered at the nuclear sites and (II) the interstitial region. The electron density and the potential are expanded into lattice harmonics (symmetry-adapted combinations of spherical harmonics) times radial functions inside the MT spheres and Fourier expansion in the interstitial region. No approximations are made on the shape of the potential and the charge density inside the spheres or in the interstitial region. In our calculations, the angular momentum expression of the lattice harmonics truncates at L = 6, and the Fourier expansion runs up to G_max_ = 14 Bohr^−1^. The wave functions inside the MT spheres are given by radial functions (which are solutions of the radial Schrödinger equations) times spherical harmonics up to l_max_ = 12, while the wave functions in the interstitial regions are described by plane wave expansions. The number of plane waves is determined by the cutoff value K_max_ (modulus of the reciprocal lattice vectors) and depends on the smallest MT sphere through the parameters R_MT_ × K_max_ = 9 (in our calculations) and R_MT_ = 2 Bohr (we used the same radius for all the atoms) [25]. For the exchange and correlation potential, we used the generalized gradient approximation Perdew–Burke–Ernzerhof (GGA-PBE) functional [26]. Although all electrons (starting from the 1s shell) are included in the calculations, the electronic states are split into two categories, core and valence states, by a cutoff energy which in the present calculations was chosen to be −10 Ry. While the core states are always treated at the fully relativistic level, the valence states can be treated at either the scalar relativistic level (no spin-orbit coupling) or at the fully relativistic level (spin-orbit coupling included). To compute the charge density in each self-consistent step, we used the tetrahedron integration method over the Brillouin zone with 1330 special k-points in the irreducible wedge corresponding to a 37 × 37 × 18 k-points mesh (25,000 k-points in the full Brillouin zone). During the self-consistency cycles, the energy, charge, and force convergence criteria were set to 0.0001 Ry, 0.0001 e^−^ and 0.5 mRy/a.u. Lattice parameters were kept fixed to the experimental values during the electronic structure calculations, while internal parameters were kept fixed or relaxed, as explained further in the text. For the relaxation of the internal parameters, we used the MSR1a method with a force tolerance of 2 mRy/a.u. (the geometry optimizations will stop when all the forces are smaller than the tolerance criteria) [25,27,28]. The effects of spin-orbit coupling were not included in our DFT calculations since, for LaPdSb, the effects are negligible. For consistent comparison at the DFT level, the spin-orbit coupling was also neglected for CePdSb. The DFT+eDMFT calculations that provide a much better description of the f states include the effects of spin-orbit coupling and the electronic correlations between the f electrons.

Charge-self-consistent density functional theory with embedded dynamical mean-field theory (DFT+eDMFT) calculations was performed with the EDMFT code [29,30,31], which implements an exact treatment of the on-site correlations of the d or f electrons while the more itinerant degrees of freedom are treated on the DFT level. In this method, it is assumed that non-local electron correlations (correlations between electrons on different atoms) are small and negligible [32]. Within this approximation, the problem of a lattice with many interacting electrons can be mapped to an Anderson impurity model, which is equivalent to an interacting site connected to a bath of non-interacting electrons (a problem that can then be solved with an impurity solver). Since the electronic band structure of the bath depends on the electronic structure of each site (therefore on the local correlations on each site), the problem has to be solved self-consistently (this is the self-consistency condition within the dynamical mean field theory) [32,33,34,35]. Another important aspect of this method is the connection between the correlated (d or f states) and non-correlated electrons (the bath), which is achieved through projection operators. These are quasi-atomic orbitals constructed from solutions of the Schrödinger equation (inside the MT sphere) projected to bands in a large hybridization window (−10 to 10 eV) with respect to the Fermi energy, in which partially screened Coulomb interactions have values of U = 6 eV and J_H_ = 1 eV for the Ce f states [29]. Since spin-orbit coupling is large enough for 4f electrons (compared with other energy scales such as the electron-electron correlations and crystal fields), it was included in the DFT+eDMFT calculations and set to 0.33 eV. The double-counting between the DFT and DMFT correlations is treated by a nominal scheme [36], with the occupancy of the quasi-atomic orbitals set to 1. The auxiliary quantum impurity problem was solved using a continuous-time quantum Monte Carlo (MC) method in the hybridization expansion (CT-HYB) [37,38], where the Ce-f orbitals were chosen as a correlated subspace in a single-site DMFT approximation. In the CT-HYB calculations, for each MC run, we employed 50 × 10^6^ MC steps. To speed up the calculations, we included only a limited number of valences for the Ce ions, in particular f^0^, f^1^, f^2,^ and f^3^. The real axis self-energy was obtained using the analytical continuation maximum entropy method for the local cumulant, as explained in [39]. During the self-consistent calculations, the position of the chemical potential was allowed to vary. The temperature was fixed at 300 K, much larger than the ferromagnetic ordering temperature (T_C_ = 17 K), such that our calculations are within the paramagnetic state.

## 3. Experimental Results: Neutron and X-ray Diffraction—Diffuse Scattering

In order to solve the conundrum of the crystal structure in CePdSb and LaPdSb, we started to refine the single-crystal neutron and X-ray data with various models proposed so far in the literature. For example, we used a model without puckering and ionic disorder corresponding to the CaIn2-type structure (Model 1 in tables) shown in Figure 1a, a model with puckering and Pd/Sb ionic disorder (Model 2 in tables), and finally, a model with puckering and ordering of the Pd and Sb ions corresponding to the ZrBeSi-type structure (Model 3 in tables) shown in Figure 1c. In all these models, the rare earths and the Pd/Sb ions form hexagonal layers alternating along the c-axis with differences only within the Pd/Sb layer. Pd and Sb ions can be statistically disordered over lattice sites with the same z coordinate, as shown in Figure 1a; they can be statistically disordered over lattice sites that split along the c axis (disordered puckering; or they can order over lattice sites that splits along the c axis (ordered puckering), as shown in Figure 1b.

Performing crystal structure refinements from scratch for the single-crystal neutron and X-ray data, we find that space group P6_3_mc (with ZrBeSi-type structure) is more suitable to explain the data instead of space group P6_3_/mmc (with CaIn2-type structure). Although the two space groups have identical selection rules for the reflections, the R1 factors characterizing the goodness of the fit are much lower for the model corresponding to the non-centrosymmetric P6_3_mc space group (which resolves the occupancy disorder by assigning to the Pd and Sb ions separate lattice sites). Space group P6_3_mc does not disturb the topology of the rare earth layers but now introduces a puckering of the Pd and Sb layers along the c-axis, see Figure 1b (within the puckered layer, Pd and Sb ions alternate around the hexagonal ring, as shown in Figure 1d). We find that the degree of puckering is more pronounced for CePdSb than for LaPdSb. A summary of the crystallographic refinements is presented in Table 1 and Table 2.

Careful data analysis of the (h, k, l) planes of diffuse scattering, see Figure 2a–c, reveal that planes with large l values show extra features, as shown by the blue arrow in Figure 2c. To understand the origin of these features, we have performed Monte Carlo simulations using a balls-and-springs model for flat (Model 1) and puckered (Model 3) Pd/Sb layers. Results of the simulations are shown in Figure 2d,f in comparison with the experimental data, Figure 2e. In particular, (h, k, 10)-layer appears quite sensitive to the puckering, which in terms of the ionic ordering translates into opposite distortions of the Pd and Sb layers along the c-axis, from the reference positions shown in Figure 1c, where no puckering exists. Similar results were obtained for the LaPdSb compound, although the structural distortion and, thus, the puckering are less pronounced, see Table 2 and Figure 3. Since the diffuse scattering is weaker in neutron scattering for LaPdSb, as shown in Figure 3b, we performed additional measurements of the diffuse scattering with X-ray scattering, shown in Figure 3e. Figure 3a,c,d,f show the corresponding Monte Carlo simulations for the structure with and without puckering. 

## 4. Theoretical Results: Microscopic Origin of the Puckering Distortion

**Figure 4:**
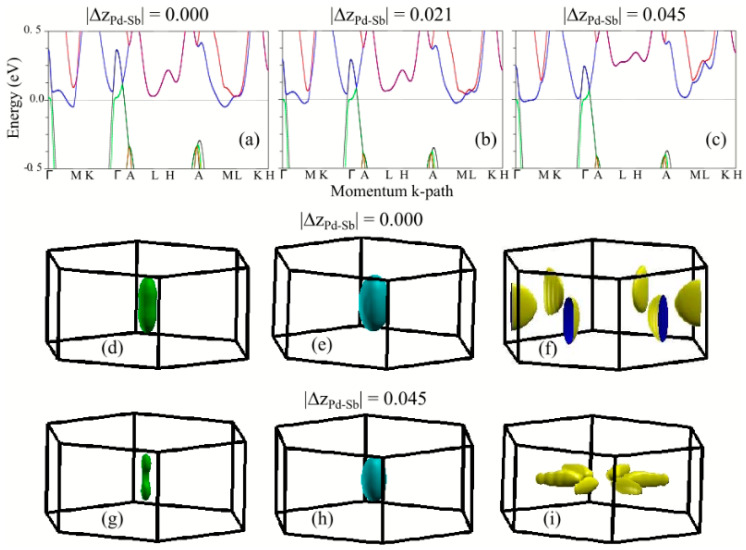
DFT electronic structure calculations for LaPdSb: panels (**a**–**c**) show the electronic band structure for three values of the puckering strength corresponding to crystal structures described in the text; panels (**d**–**f**) show the Fermi surfaces corresponding to the band structure from panel (**a**) and panels (**g**–**i**) show the Fermi surfaces corresponding to the band structure from panel (**c**).

To elucidate the microscopic origin of the puckering, we carried out nonmagnetic DFT electronic structure calculations for the LaPdSb and CePdSb compounds and DFT+eDMFT paramagnetic calculations for the CePdSb at room temperature. To probe the effects of increasing the puckering on the band structure, we used reference crystal structures with no puckering (see the corresponding band in Figure 4a), experimental crystal structures with medium puckering (see the corresponding band in Figure 4b) and crystal structures with fully relaxed internal coordinates, corresponding to a larger puckering (see the corresponding band in Figure 4c). In all these calculations, the lattice parameters were kept fixed to the experimental values. Electronic band structure calculations for the LaPdSb compound without puckering reveal three bands crossing the Fermi energy, two of them around the Γ point and one around the M point, as shown in Figure 4a. These bands translate into three Fermi surface pockets, shown in Figure 4d–f, with two hole pockets around the Γ point and one electron pocket around the M point. Upon introducing puckering and ionic order in the Pd and Sb layers, we see significant changes for the bands around the M point, as shown in Figure 4b,c. For example, large puckering leads to the electron pocket disappearing from the M point while another pocket reappears along the M-Γ path with a slightly different topology, as shown in Figure 4f,i. The two-hole pockets have the same topology but a decreased surface, see Figure 4d,e,g,h.

**Figure 5:**
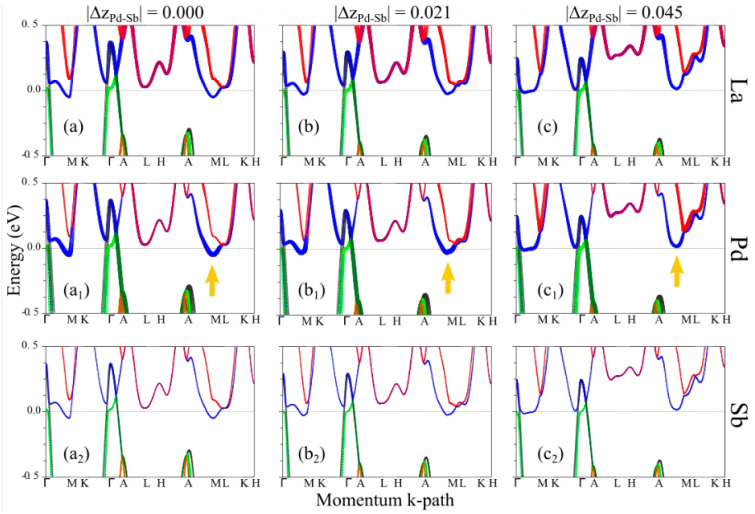
Fat band representation of the DFT electronic structure calculations for LaPdSb: panels (**a**,**a_1_**,**a_2_**) show the atomic contributions to the bands for the crystal with no puckering; panels (**b**,**b_1_**,**b_2_**) together with panels (**c**,**c_1_**,**c_2_**) show the atomic contributions to the bands for two distinct crystal structures with puckering as described in the text; the yellow arrow shows the dependence of the electron pocket Pd contribution at M point versus puckering.

To obtain a deeper insight into the electronic structure around the M point, in Figure 5, we show the electronic band structure with the atomic projections (fat band representation [25,40,41]). Independently of the puckering strength, we see that bands around the Fermi energy have contributions mostly from La and Pd ions (moreover, inspecting further the band character, we find mostly La-d and Pd-d orbital character in this energy range). In addition, we learn that while increasing puckering strength, the contribution of La ions around the M point does not change too much (see Figure 5a–c), while the contribution of the Pd ions is decreasing (see Figure 5a_1_–c_1_), Pd having the smallest contribution when the bands are above the Fermi energy (in other words, when the electron pocket at the M point does not exist anymore).

The total energy of the ground state decreases with increasing the puckering strength showing that the puckering renders the crystal structure more stable. The crystal structure with the largest puckering is ~15 meV/f.u. lower than the crystal structure with no puckering. 

**Figure 6:**
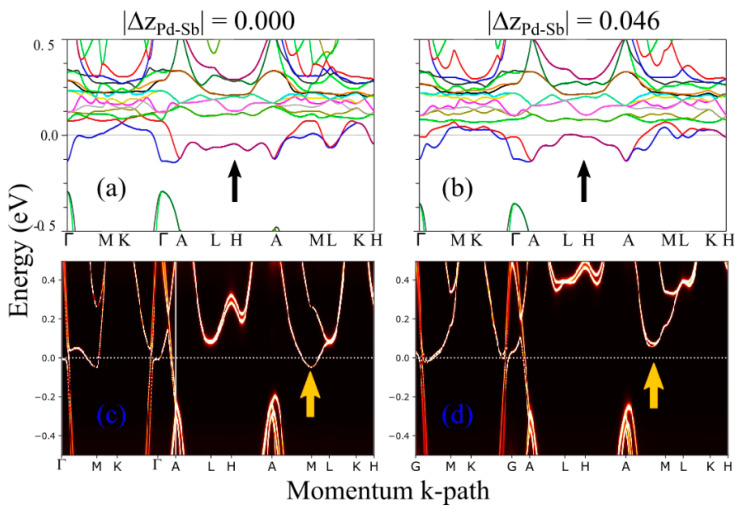
Electronic structure calculations for CePdSb: DFT calculations for a structure with no puckering, panel (**a**) versus a structure with puckering, panels (**b**–**d**) show the spectral functions obtained from the DFT+eDMFT calculations, for the same structures used in panels (**a**,**b**); the black and yellow arrows point to the most significant changes occurring in the electronic structures versus puckering; in all calculations, we used the experimental crystal structures.

Similar calculations have been performed for the CePdSb compound; see Figure 6a,b. By comparing DFT calculations for CePdSb and LaPdSb, we observe a significantly larger number of states above the Fermi energy for the CePdSb compound due to the f states which in nonmagnetic DFT approximation are treated as weakly correlated valence states; although, in reality, the f electrons are strongly correlated. As a consequence of the hybridization between the Ce f states with the other Pd- and Sb-s, p, d states, the topology of the bands crossing the Fermi energy is different from the topology of the LaPdSb compound, see Figure 4a,b compared to Figure 6a,b. Despite these differences, we still find a systematic trend in the electronic band structure, such as changes (in the form of band shifts) appearing with increasing puckering. In addition, the structure with the largest puckering has the lowest total energy (~14 meV/f.u) compared to the refence structure with no puckering, in line with trends in LaPdSb. Although nonmagnetic DFT captures some trends in the Ce compound qualitatively, it is well known that nonmagnetic calculations, without properly treating the strong on-site Coulomb repulsion (correlations) between the Ce–f electrons and the spin-orbit coupling, have severe shortcomings when applied to studying f electrons system [42,43,44,45,46].

**Figure 7:**
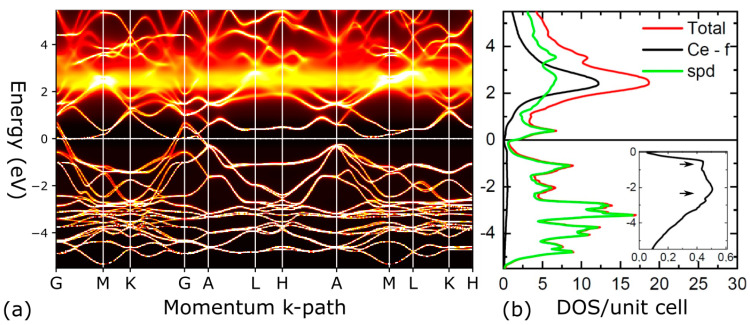
DFT+eDMFT spectral function and density of states for CePdSb: Spectral function with the −5.5 to 5.5 eV, panel (**a**) and density of states panel (**b**); the inset in panel (**b**) shows the density of states for the Ce–f states below the Fermi energy and the arrows points toward a two peaks structure in the density of states.

These shortcomings can partially be remedied by dynamical mean-field theory (DMFT) in combination with DFT, which generally leads to a better description of the electronic structure of correlated materials [29,30,31,32]. Thus, we turn our attention toward DFT+eDMFT methods, and we perform additional calculations for CePdSb. Spectral functions, shown in Figure 6c,d for CePdSb, reveal that by properly describing the f electrons as correlated, we recover a band structure that is similar to LaPdSb. This implies that the f states do not hybridize strong enough (for small energies around the Fermi energy) with the other s, p, d states to drastically change the topology of the bands around the Fermi energy in comparison with the non-correlated LaPdSb compound. There is strong hybridization between the f states and the other non-correlated states away from the Fermi energy, but the smallest hybridization is found around the Fermi energy. Within the DFT+eDMFT, the experimental puckered crystal structure, with the corresponding spectral function shown in Figure 6d, has a lower energy than the non-puckered crystal structure, with the corresponding spectral function shown in Figure 6c. In Figure 7, we show the spectral function for a larger energy window together with the density of states for the correlated Ce-f states and non-correlated s, p, d states coming from the La, Pd, and Ce ions. 

## 5. Discussion

Neutron and X-ray diffraction techniques have been used to study the crystal structures and diffuse scattering in LaPdSb and CePdSb intermetallic compounds. These experimental techniques were complemented by first-principles techniques such as DFT and DFT+eDMFT. Based on our study, we find that (I) LaPdSb and CePdSb compounds are best described by the hexagonal ZrBeSi-type structure (with the non-centrosymmetric space group P6_3_mc) where Pd and Sb ions order within puckered layers while no distortion exists in the rare earth layers. Total energy calculations are consistent with the experimental results and imply the stability of the puckering model; (II) a strong correspondence exists between the puckering and the Pd bands character around the M point for the LaPdSb compound; (III) there is also a correspondence between a decreasing trend of the Pd band character and lowering the total energy, thus demonstrating that the energetically more stable structure has less Pd band contribution around the M point; (IV) a second correspondence exists between the structural puckering and the electronic band structure/pockets around the Fermi energy. Furthermore, a fat band representation of the electronic band structure provides us with insights into the microscopic origin of the structural puckering, suggesting that Pd-d and La-d hybridization could be responsible for the stability of the puckering within the crystal structure.

By treating the f-electrons as correlated, the spectral functions for CePdSb show a similar band structure to LaPdSb, as expected, since both compounds show experimentally the same structural features. The f orbital occupation, n_f_ = 1, obtained from DFT+eDMFT calculations is consistent with the published 3d X-ray photoemission spectroscopy [10,11]. There is also a qualitative agreement between the total density of states shown in Figure 7 with the published X-ray photoemission spectroscopy spectra shown in Figure 2 from Ref. [10] and the Ce-4f density of states shown in Figure 7b with the published resonance photoemission spectra shown in Figure 3 and Figure 4 from Ref. [11]. Our DFT calculations are also consistent with other DFT calculations performed in the literature and presented only as the density of states [10,11,12]. The theoretical methods used in this paper have been successfully applied, so far, to weakly correlated materials [47,48,49,50,51,52,53] and strongly correlated materials [28,29,30,32,42,54,55,56,57,58,59,60,61]. The importance of spin-orbit coupling to the physical properties has also been demonstrated for the f electrons [29,42,43,44,45,46,60,61].

A simple comparison of the Fermi surfaces with the diffuse scattering features implies the possibility of a correspondence between the Fermi surface pockets at the M point and the features present in the diffuse scattering around the same M point; thus, our results could be relevant to the imaging of the Fermi surface by the diffuse scattering method [62]. In addition, since the shape of the Fermi surface is very important to the electric, magnetic and thermal properties of materials, measurements of the Fermi surface are of great interest to validate the theoretical predictions. Even though structural distortions are small, we show their relevance to the electronic properties such as band structure and Fermi surfaces. Another example of a material where small lattice distortions are relevant to the electronic properties is FeSb_2_, which is a material with the highest known thermoelectric power [63].

Materials developing ferromagnetic ground states, intermetallic compounds based on rare-earth elements or rare-earth and transition metal elements, are finding applications in many fields, from spintronic devices to electronic devices to biomedicine [64,65,66,67,68,69]. An important aspect characterizing the ferromagnetic materials is the Curie temperature, which varies drastically between classes of materials or even for materials within the same class, depending on the microscopic ingredients giving rise to the ferromagnetic ground state. Intermetallic compounds based on transition metals have a larger Curie temperature, while those based only on rare-earth have smaller ordering temperatures [70,71,72,73,74]. CePdSb itself has a small Curie temperature compared with the requirements for practical applications, but it has one of the largest ordering temperatures of its class. At the same time, a ferromagnetic ground state combined with a non-centrosymmetric space group gives rise to a spin-polarized band [75]. LaPdSb has a large power factor and a large dimensionless figure of merit around room temperature [13,14]. Thus, studies on CePdSb and LaPdSb could be used to advance our understanding of ferromagnetic ground states in rare-earth-based intermetallics compounds, of spin-polarized bands in magnetic and non-magnetic compounds, of thermoelectric mechanisms, etc.

## 6. Conclusions

In conclusion, using a combination of novel experimental and theoretical techniques, we solve the conundrum related to the crystal structure models in the LaPdSb and CePdSb intermetallic compounds, and we find the best structural model given by the non-centrosymmetric P6_3_mc space group. We demonstrate that, using diffuse scattering, we can distinguish between crystallographic models where standard experimental techniques give indistinguishable results. First principle calculations reinforce our experimental findings and show that the crystal structure described by the P6_3_mc space group is energetically more stable than the previously assigned space groups. In addition, theoretical calculations show a strong dependence between the electronic properties and structural distortions, thus indicating an interesting electronic-structural interplay that associates diffuse scattering with electronic properties. DFT+eDMFT calculations within the paramagnetic state are validated by published X-ray photoemission spectroscopy measurements, displaying the predictive powers of the method for the electronic properties of correlated materials. Our results can have important implications for the microscopic understanding of the electronic-structural interplay in RPdSb class of materials, thus on the multifunctional physical properties of these materials.

## Figures and Tables

**Table 1:** Summary of crystallographic refinements for LaPdSb.

	Model 1 Flat Pd/Sb	Model 2 Split Pd/Sb	Model 3 Puckered Pd/Sb
LaPdSb			
Space group	P6_3_/mmc	P6_3_/mmc	P6_3_mc
a = b [Å]	4.6134 (10)	4.6134 (10)	4.6134 (10)
c [Å]	8.1470 (20)	8.1470 (20)	8.1470 (20)
V [Å^3^]	150.17 (6)	150.17 (6)	150.17 (6)
La (x, y, z)	(0, 0, 0)	(0, 0, 0)	(0, 0, 0) *
U_11_ = U_22_[Å^2^]	0.0088 (1)	0.00893 (4)	0.00898 (2)
U_33_ [Å^2^]	0.0072 (1)	0.00767 (4)	0.00746 (3)
Pd (x, y, z)	(1/3, 2/3, 3/4)	(1/3, 2/3, 0.7380 (5))	(1/3, 2/3, 0.7429 (4))
U_11_ = U_22_[Å^2^]	0.0073 (1)	0.00725 (3)	0.00822 (3)
U_33_ [Å^2^]	0.0240 (3)	0.0170 (1)	0.01155 (6)
Sb (x, y, z)	(2/3, 1/3, 3/4)	(2/3, 1/3, 0.7591 (5))	(2/3, 1/3, 0.76424 (6))
U_11_ = U_22_[Å^2^]	0.0073 (1) **	0.00725 (3) **	0.00602 (2)
U_33_ [Å^2^]	0.0240 (3) **	0.0170 (1) **	0.02594 (2)
NRefs (I > 3σ/all) Neutron X-ray ***	9273/2687 1278/314	9273/2687 1278/314	9273/2687 2359/646
R_1_/wR_2_ (I > 3σ)	23.07/64.69	8.17/22.05	6.49/15.54
R_1_/wR_2_ (all)	24.67/64.73	9.39/22.45	7.81/16.06
GooF (I > 3σ/all)	19.97/17.64	5.90/5.30	4.23/3.85

* Origin fixed, ** Constrained to be equal to Pd, *** X-ray data were merged.

**Table 2:** Summary of crystallographic refinements for CePdSb.

	Model 1 Flat Pd/Sb	Model 2 Split Pd/Sb	Model 3 Puckered Pd/Sb
CePdSb			
Space group	P6_3_/mmc	P6_3_/mmc	P6_3_mc
a = b [Å]	4.6071 (7)	4.6071 (7)	4.6071 (7)
c [Å]	7.9371 (16)	7.9371 (16)	7.9371 (16)
V [Å^3^]	145.90 (4)	145.90 (4)	145.90 (4)
Ce (x, y, z)	(0, 0, 0)	(0, 0, 0)	(0, 0, 0) *
U_11_ = U_22_[Å^2^]	0.0098 (4)	0.0103 (1)	0.00974 (8)
U_33_ [Å^2^]	0.0053 (4)	0.0057 (1)	0.00687 (8)
Pd (x, y, z)	(1/3, 2/3, 3/4)	(1/3, 2/3, 0.7400 (2))	(1/3, 2/3, 0.7166 (1))
U_11_ = U_22_[Å^2^]	0.0069 (3)	0.00719 (7)	0.0069 (1)
U_33_ [Å^2^]	0.060 (1)	0.01262 (5)	0.0354 (3)
Sb (x, y, z)	(2/3, 1/3, 3/4)	(2/3, 1/3, 0.7824 (3))	(2/3, 1/3, 0.76295 (7))
U_11_ = U_22_[Å^2^]	0.0069 (3) **	0.00719 (7) **	0.0072 (1)
U_33_ [Å^2^]	0.060 (1) **	0.01262 (5) **	0.0117 (1)
NRefs (I > 3σ/all)	6216/6	6126/6	6216/6
R_1_/wR_2_ (I > 3σ)	41.90/82.02	11.61/28.90	8.03/19.11
R_1_/wR_2_ (all)	41.91/82.02	11.62/28.95	8.04/19.18
GooF (I > 3σ/all)	31.47/31.45	8.50/8.50	5.58/5.59

* Origin fixed, ** Constrained to be equal to Pd.

## Data Availability

The data used to support the findings of this study are available from the corresponding authors upon request.
